# Air monitoring of aromatic hydrocarbons during automobile spray painting for developing change schedule of respirator cartridges

**DOI:** 10.1186/2052-336X-12-41

**Published:** 2014-01-27

**Authors:** Mehdi Jahangiri, Javad Adl, Seyed Jamaleddin Shahtaheri, Hossein Kakooe, Abbas Rahimi Forushani, Mohammad Reza Ganjali

**Affiliations:** 1 Department of Occupational Health, School of Health and Nutrition, Shiraz University of Medical Sciences, Shiraz, Iran; 2 Department of Occupational Health, School of Public Health, Tehran University of Medical Sciences, Tehran, Iran; 3 Departments of Occupational Health, School of Public Health, and Institute for Environmental Research, Tehran University of Medical Sciences, Tehran, Iran; 4 Department of Biostatistics, School of Public Health, Tehran University of Medical Sciences, Tehran, Iran; 5 Center of Excellence in Electrochemistry, Faculty of Chemistry, University of Tehran, Tehran, Iran

**Keywords:** Respirator, Cartridge change schedule, Air monitoring, Spray painting

## Abstract

In the absence of End of Service Life Indicator (ESLI), a cartridge change schedule should be established for ensuring that cartridges are changed before their end of service life. Factors effecting service life of cartridges were evaluated, including the amount of atmospheric contamination with aromatic hydrocarbon vapors in the workplace, temperature, and relative humidity of the air. A new change schedule was established based on comparing the results of air monitoring and workplace conditions, laboratory experiment, and the NIOSH MultiVapor software. Spray painters were being exposed to aromatic hydrocarbons in a range exceeding occupational exposure limits. The cartridge change schedule was not effective and could no longer provide adequate protection against organic contaminants for sprayers. Change schedules for respirator cartridges should be reduced from 16–24 hours to 4 hours. NIOSH’s service life software program could be applied to developing cartridge change schedules.

## Introduction

In the car manufacturing industry, spray painters should wear suitable types of respirators throughout the entire spraying process to minimize their exposure to volatile organic compounds (VOCs). This may either be the primary exposure control - especially necessary when spraying is carried out in poorly ventilated conditions - or a supplementary exposure control in conjunction with other measures.

Most air-purifying respirator cartridges for organic vapor contain a packed bed of activated carbon granules. The service life (breakthrough time) of this absorbent depends on different parameters, including cartridge specifications (amount of sorbent in cartridge, the activated carbon’s properties, and bed geometry) and its conditions of use (concentration of workplace atmospheric VOCs, relative humidity and temperature, plus workers' breathing rates) [1,2].

Based on the Occupational Safety and Health Administration (OSHA) respiratory protection standard [3], if there is no end-of-service-life indicator (ESLI) for canisters and cartridges, a change schedule, based on objective information which will ensure that canisters and cartridges are changed before the end of their service life, must be implemented. OSHA suggests three valid ways to estimate the cartridge's service life and establish a change schedule for them: conducting experimental tests, using the manufacturer's recommendation, and using a mathematical model [4].

Several studies have been done for the evaluation of efficiency and developing models for estimation of the service life of organic vapor cartridges. Gary O. Nelson et al. determined the service life for organic vapor cartridges under a wide variety of air flow concentrations [5], air flow, humidity and temperatures [6], and solvent vapor concentrations [7]. They also presented an empirical expression for estimating the service life of cartridges [8].

Different models and equations have been presented in literature for estimating the service life of cartridges, including the D/R, Mecklenburg, Wheeler, Yoon and Wood equations [9].

The most common model used by OSHA, and some manufacturers, is the Wood math model [10], which was presented for estimating the service lives of both single organic vapor [11] and multiple organic vapor cartridges [12] at all possible humidity values.

However, most of the above studies were performed for estimation of service life of respirator cartridges and very few studies looked at the practical aspects of establishing change schedules in workplaces. Furthermore, developing a cartridge change schedule is a new exercise for most respirator users. Because standard approaches to setting a change schedule have neither been developed nor validated, there is uncertainty about their efficacy [13].

The aim of the present work was: (1) evaluation of efficacy of existing change schedules in the study workshop; (2) air monitoring of aromatic hydrocarbons in the automobile spray painting workshop; and (3) developing a cartridge change schedule based on the results of air monitoring and workplace conditions. The practical goal of this study was the introduction of a change schedule for workers who were using air purifying respirators to protect themselves against VOCs during spray painting. They would thus be able to predict when cartridges no longer provided adequate protection.

## Material and methods

### Evaluation of efficacy of existing change schedule

**Figure 1:**
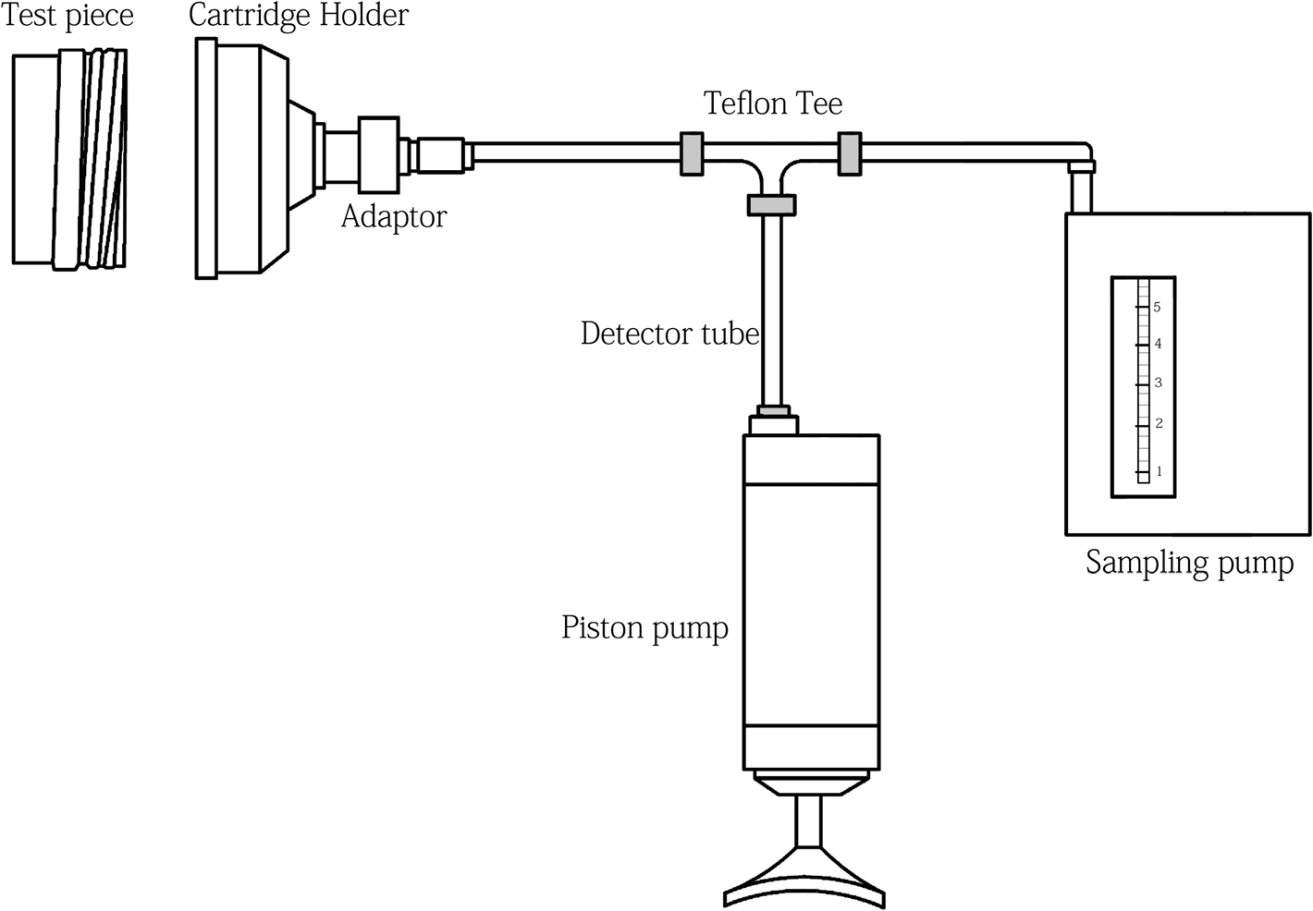
Schematic diagram of the set-up for field testing of cartridge change-out schedule.

The efficacy of the existing change schedule of air-purifying respirator cartridges used in the automobile spray painting workshop was investigated using a procedure recommended by OSHA [3]. The apparatus was used for this purpose, was made of detector tubes (as direct reading instruments), a leak free cartridge holder, adaptors, inert tubing and Teflon tee, sampling tube, and a sampling pump, as shown in Figure 1. For testing the efficacy of the existing change schedule, immediately before changing the cartridges being used in the workplace, the breakthrough of benzene from 10 randomly selected cartridges was tested as air was passing through them. If breakthrough of benzene was detected above the occupational exposure limit, it would show that the existing industry schedule for changing the cartridges is not efficient and a new cartridge change schedule should be developed.

### Air monitoring of aromatic hydrocarbons

The concentrations of benzene, toluene, and xylenes (BTX) were measured, both as a representative set of VOCs and as the most common organic compounds found in spray painting workshops. We collected 24 personal air samples from painters’ breathing zones (as a similar exposure group) over six consecutive working days. This was done at times of peak exposure.

The frequency and duration of exposure of employees wearing respirators was also determined and the cumulative personal solvent exposure over an eight-hour work shift was calculated. Data from lunch breaks were not included as exposure time when calculating the air concentration. Time that a painter was not actually present in the spraying booth, but was instead engaged in the preparation of paints was considered as exposure time.

A standard analytical procedure (National Institute for Occupational Safety and Health-NIOSH 1500/1501) was used to analyze the air samples. Briefly, the organic chemicals were desorbed from the charcoal using CS2 and analyzed by gas chromatography (Shimadzu) operated in a split mode. Twenty-four major chromatogram peaks were identified in daily samples based on a comparison of retention times and mass spectra to peaks from a calibration standard.

On each working day, one field blank was collected along with the two daily samples and was analyzed with the same procedure as for the air samples.

The worst-case concentration of contaminants was calculated by adding the average concentration over six working days to the standard deviation, and the result was used for cartridge service life estimation.

In addition to determining the concentration of contaminants, other workplace conditions that could influence the service life of a cartridge, including relative humidity and temperature of the workplace atmosphere, were measured with the aid of a Digital Humidity/Temperature Meter Model: MTH-1361 over six working days, and their maximum values were recorded as worst-case conditions for cartridge service life estimation.

Workers' breathing rates were estimated based on the type of work that operators were doing in the spray painting booth and this amount was used as total airflow passed through the respirator cartridge.

### Cartridge testing and change schedule

The cartridge change schedule was determined by a laboratory experiment and the results were compared with those of the NIOSH MultiVapor software for estimation of the service life of cartridges.

#### *Determination of service life based on the laboratory experiment*

To determine the service life of a cartridge by laboratory experiment, four as-received respirator cartridges (3 M 6001) were exposed to the atmospheric contaminants found in the workshop, including benzene, toluene, and xylenes, both individually and mixed together.

For this purpose, a gas or vapor stream was blended with clean air to the desired concentration and passed through the cartridge at a specified flow rate until the desired fraction of the upstream concentration is achieved downstream of the cartridge. The length of time taken for a gas or vapor to saturate sorbent material in the cartridge, under a given set of environmental variables, was considered as the breakthrough time.

The breakthrough time may be expressed in minutes at a given flow rate, challenge concentration, or percent breakthrough. The air temperature and relative humidity were adjusted according to the workplace atmospheric conditions.

**Figure 2:**
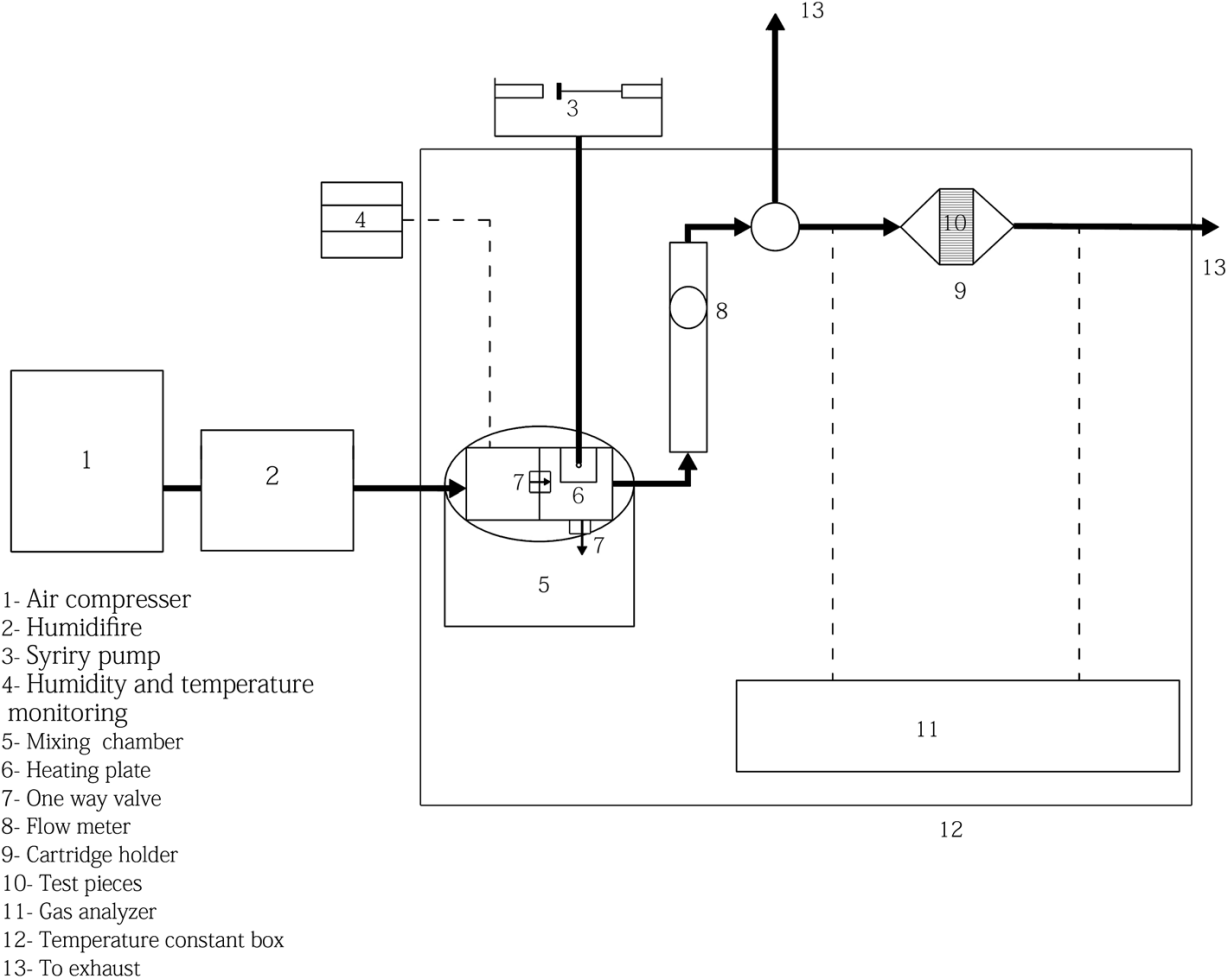
A schematic diagram of the apparatus used for measuring the cartridge breakthrough time.

Figure 2 shows the schematic diagram of the apparatus designed for measuring the breakthrough time of the respirator cartridge. The main part of this apparatus was the mixing chamber, which was made of PTFE and consisted of three parts. The first part was equipped with temperature and relative humidity sensors (TC4Y-14R, Autonics, Korea), The second part accommodated a heating plate (item 6) for vaporizing the solvent as it entered from the syringe pump (model HX-901A, item 3). After humidification, the air (item 2) passed through the first part to the second, mixed with vaporized solvent, and then entered into the third part (item 5, the main part of mixing chamber).

These parts were clamped together with an o-ring to provide a leak-proof fit, and two one-way valves (items 7) between the parts prevented back pressure inside the chamber.

Inlet air was provided by an air compressor, filtered for organics and particulates, and passed through a humidifier to control and maintain a constant humidity in the mixing chamber. Temperature and relative humidity were adjusted according to the worst-case conditions of workplace by means of a humidity controller (SU-503B, Samwon Eng, Korea).

The mixing chamber output was directed into a cartridge holder where the test piece was fixed. At the start of run the mixing chamber output was bypassed to vent (13). After adjusting both the temperature and humidity, and after obtaining a steady state concentration based on the desired values (which normally took 30 min), the bypass valve was shut, the air stream was allowed into the main line, and the test started.

The airflow was passed through the respirator cartridge at a flow rate similar to the average breathing rate of painters working in a painting booth and wearing a respirator.

#### *Vapor generation and concentration measurement*

All substances used for vapor generation in the present study were of GC grade (Merck, Germany). By controlling the injection rate of the syringe pump, the organic solvent vapor concentration was set in the range of worst case concentrations of contaminants found in spray painting booths. The variation of these concentrations was less than 10% of the desired value. Experiments in which a sudden decrease in concentration was observed were repeated.

The vapor concentration of individual contaminants was measured both upstream and downstream of the cartridge, using a gas analyzer equipped with a Photo Ionization Detector (Ion Sciences Co., UK). The accuracy of the gas analyzer was checked by a Gas Chromatograph (Varian model CP-3800) equipped with a hydrogen flame ionization detector (FID).

When testing cartridges with mixtures of organic solvents, two gaseous samples - one upstream and one downstream of the cartridge - were collected every five minutes and were directly injected into the gas chromatograph equipped with a FID.

#### *Breakthrough time measurement*

The interval between the start of test and the time when the vapor concentration downstream of the cartridge reached 50% of the Threshold Limit Value (TLV of the American Conference of Governmental Industrial Hygienists – ACGIH- 8-hour TWA) [14] for one particular substance was recorded as the breakthrough time in relation to the vapor concentration and humidity. For each substance, the shortest recorded time was considered as the breakthrough time.

#### *Estimation of service life based on NIOSH MultiVapor software*

Software programs exist for the estimation of cartridge service life. OSHA has developed one (Advisor Genius) to predict the service life of organic vapor respirator cartridges when used as protection against single contaminants [15]. Most available software programs to calculate breakthrough time are based on exposure to a single contaminant and are strongly influenced by high humidity. However, NIOSH recently developed a software program - MultiVapor [16] - which can be used for both single and multi vapor contaminants. In this study, in addition to laboratory experiment, service lives of used cartridges were also estimated using NIOSH MultiVapor software for both single and multi contaminants. After calculating the breakthrough time for each of the mixture’s components, the breakthrough time of xylene (m-xylene) was considered for setting out a change schedule, as it had the shortest breakthrough time in the mixture [3].

Workplace conditions, including concentration of contaminants, relative humidity, temperature, and workers' breathing rates, all of which had been reproduced in the laboratory experiments, were analyzed using the MultiVapor software.

### Evaluation of efficacy of developed change schedule (Field testing)

An estimated change schedule was evaluated and verified using the procedures described in step 2.1. At this stage, if organic vapors were not detected in the samples (used cartridges), then significant breakthrough would not have occurred. Thus the change schedule would be confirmed and cartridges could be changed according to it. If breakthrough had occurred, then the designed cartridge change schedule would have to be shortened (usually by one hour) and the test would have to be repeated.

## Results

In the spray booth, five sprayers worked 10 hours a day and six days per week. Painters wore half-face respirators to protect themselves against organic vapor for eight hours.

Each cartridge contained 46.39 g of sorbent. The manufacturer reported a carbon micro pore volume of 0.533 m^3^/g and an average carbon granule diameter of 0.15 cm. The manufacturer reported the carbon bed dimensions to be 8 cm diameter and 2.2 cm depth.

The existing schedule for changing the cartridges in the workshop studied ranged from two to three working days (16–24 hours) and was based on either the painter's reliance on odor threshold of contaminants or difficulty in breathing. To evaluate the efficacy of this cartridge change schedule a breakthrough of benzene was tested in the spray booths, just before the usual cartridge change. This was done for 10 cartridges using the method described in section Material and methods.

A benzene breakthrough was observed in seven cartridges, suggesting that the existing cartridge change schedule in the spray booth was not efficient and painters would be exposed to organic vapors, especially as existing engineering controls were not working properly. The change schedule should therefore be revised.

### Workplace air monitoring and characteristics

Table 1 shows the results of air monitoring and other workplace characteristics which are required for developing a cartridge change schedule, including air humidity and temperature.

**Table 1:** Workplace air monitoring and other characteristics of workplace

**Parameter**		**n**	**M ± SD**	**TLV**	**MD**	**t**	Sig. (2- tailed)^*^	Worst case condition^**^
Concentration (ppm)	Benzene	24	36.22 ± 21.22	0.5	36.05	5.09	0.001	58
	Toluene		111.33 ± 61.79	20	91.44	4.42	0.002	174
	Xylenes		312.33 ± 91.33	100	212.92	6.99	0.000	404
Temperature (C)		6	20.83 ± 0.68	-	-	-	-	22
Relative humidity (%)		6	54.5 ± 0.5	-	-	-	-	55

M ± SD; Mean ± Standard Deviation, MD; mean Difference, TLV; Threshold Limit Value ACGIH (8-hour TWA).

*One-sample Test.

**Worst case conditions were calculated by adding mean measured values to their standard deviation.

The results of air monitoring showed that the concentrations of organic solvent vapors were in the ranges of 36.2 ±21.2, 111.3 ± 61.7 and 312.3 ± 91.3 ppm for benzene, toluene and xylenes, respectively. This is higher than the ACGIH 8-h TLV–TWA (P < 0.05). This finding is similar to Vitali et al.’s study in which the concentrations of benzene found were at levels close to or higher than the 8-h TLV–TWA in all car painting shops studied [17].

The worst case concentration of organic solvent vapors in the spraying booth was calculated by adding the average measured concentration to its standard deviation, and this was used to estimate cartridge service life. The worst case concentrations of these solvents were 58, 174 and 404 ppm for benzene, toluene and xylenes, which are respectively 115.5, 8.7 and 4 times higher than their ACGIH TLV.

The temperature and relative humidity of the air in the spraying booths were 20.83 ± 0.68°C and 54.5 ± 0.5% percent respectively, which are the worst case conditions of the workplace.

Air passed through the respirator cartridge at a flow rate of 37 l/min, which was the average breathing rate for operators doing moderate to heavy work in the painting booths and wearing their respirators.

### Cartridge testing and service life estimation

Table 2 shows the estimation of cartridge service life by both the experiment and the use of NIOSH MultiVapor software. According to our tests, using the extreme conditions of the workplace studied, cartridges resisted for a maximum of 22.58, 14.62 and 8.56 hours for benzene, toluene and xylenes respectively (for single contaminants). But in the real situation when there is a mixture of these contaminants, the cartridges’ maximum breakthrough time was 4.35 hours. As Table 2 shows, NIOSH software estimated a longer service life than the experimental values, with the MultiVapor software exhibiting relative errors of 9.71, 9.29, 10.28, and 12.62 percent respectively for benzene, toluene, xylenes and a mixture of these contaminants.

**Table 2:** **Estimation of cartridge service life by experiment, manufacturer**'s **calculation and NIOSH MultiVapor software**

**Test conditions**		**Challenge concentration ppm**	**Breakthrough criteria (ppm) (50% TLV)**	**Breakthrough time (min)**		**Relative error of NIOSH MultiVapor software (Percent)**
**Test agent**						
				**Experiment**	**NIOSH MultiVapor software**	
Single vapors	Benzene	58	0.25	1355.3	1487	9.71
	Toluene	174	10	877.2	958.7	9.29
	Xylenes*	404	50	514.4	9.32	10.28
Mixtures	Benzene, Toluene and Xylenes		0.25	261.3	294.3	12.62

*Estimation for xylenes has been done based on the primary component of m-xylene, Atmospheric Pressure (atm): 1.

## Discussion

The breakthrough time for organic vapors in this study was estimated at four hours. However, the US OSHA standard for benzene [18] requires that organic vapor cartridges be replaced at expiration of their service life or at the beginning of each shift in which the cartridges are used, whichever comes first. Thus, even if engineering controls, such as booth ventilation, were improved, then based on the existing industry schedule the organic vapor cartridges would not be changed. In other words, cartridges must never be used for longer than one shift for benzene even if the estimated service life is longer than a shift or the cartridge is used for only part of a shift.

Although there is some uncertainty and there are problems associated with cartridge change schedules, it is believed that the uncertainties of change schedule present less of a public health problem than would the continued reliance on warning properties [19].

To overcome these uncertainties and prevent inadequate change schedules that could result in chronic overexposure, we verified the change schedule that we had developed. At the end of estimated service life (four hours) and immediately before changing the cartridges, we again tested 10 cartridges for benzene breakthroughs in the spraying booths and no benzene breakthroughs were detected. This reaffirmed the newly established change schedule and cartridges could be changed according to it. However, change schedules should be re-evaluated on a systematic and frequent basis, as well as when workplace conditions change.

The best change schedules are ideally based on cartridge breakthrough tests under worst case conditions of contaminant concentration, humidity, temperature, and air flow through the filter element [3], but in practice this method is difficult and time consuming. As Table 2 shows there is 15% difference between the experimental measured breakthrough time and its estimated value from NIOSH MultiVapor software. Therefore in practice, NIOSH’s service life software program could be applied to developing cartridge change schedules if the estimated service life was reduced by a safety factor of at least 15%.

In the workplace under study, the service life (multiple contaminants) was estimated to be less than one shift, so the cartridges must be disposed of according to the established change schedule, which is every four hours.

Reliance on odor thresholds and other warning properties have long been recognized as problematic and could not be considered solely as criteria for replacing cartridges (NIOSH 2004). This is because:

1. Warning properties rely upon fallible human senses.

2. There is typically a wide variation of odor threshold in the general population with respect to the detection of odors.

3. Humans’ odor thresholds can shift due to extended low exposures and some illnesses, like a simple cold.

When the odor threshold of a compound is greater than its TLV, overexposure of a respirator user is possible since breakthrough may not be detected. For benzene, the odor threshold is about 4.7 ppm [20] or approximately 10 times its occupational exposure limit in Iran (0.5 ppm). So benzene has a poor warning property and its odor cannot be considered a safe criterion for warning of its breakthrough and the need to change the cartridge. The odor threshold for xylene is 1 ppm of air. Because this value is below the current ACGIH TLV–TWA of 20 ppm [14], xylene is considered to have adequate warning properties. In the case of toluene, in spite of having an odor threshold of 2.49 ppm - below its current permissible exposure limit (PEL) of 100 ppm there are wide variations in its odor threshold and therefore its warning property is not considered reliable [21].

Relying on odor thresholds and other warning properties as the primary means to determine the time to change the chemical cartridge is not effective and is rejected in OSHA’s revised respiratory protection code. Instead OSHA requires that a change schedule be established that identifies how long a chemical cartridge can be used in a particular workplace before being replaced. OSHA also states that chemical cartridges could be used for chemicals with poor warning properties if an effective change schedule is established.

As mentioned earlier, the best way to control the degree of exposure to hydrocarbon aromatics in a spray booth is to eliminate the use of hazardous substances, or to use less hazardous substances. Replacing spray painting with rolling or brushing systems, or isolating the spray painting using a fully automated process, can also reduce exposure. Where this is not practicable, engineering controls such as ventilation systems should be considered. The ventilation system in spray booths should provide a continuous, uniform and evenly distributed air flow of acceptable quality throughout the spray painting area, as well as a sufficient air velocity at all points within the operators’ breathing zones.

Using air purifying respirators is generally considered to be a supplemental control measure. However, it is sometimes the main control measure prior to the implementation of environmental controls, or when there is a ventilation system failure that could potentially result in over exposure to organic vapors.

Unfortunately, in the workplace studied, the ventilation system was not able to control the painters’ degree of exposure; they therefore had to rely on using respirators for their protection. Proper usage and replacement of cartridges before the end of their service life is very important for protection during work in a spray booth.

## Conclusion

Based on the schedule established in this study, change schedules of respirator cartridges should be reduced from 16–24 hours to 4 hours.

Cartridge change schedules for air-purifying respirators must be established based on the results of workplace air monitoring and other use conditions of cartridges. They should not rely on odor and other warning properties of contaminants.

Furthermore, this study also showed that NIOSH’s service life software program could be applied to developing cartridge change schedules if the estimated service life was reduced by a safety factor of at least 15%.

## Competing interests

The authors declare that they have no competing interests.

## Authors’ contributions

The overall implementation of this study including design, experiments and data analysis, and manuscript preparation were the results of efforts by first three authors. Other authors have helped in experimental design and data analysis. All authors read and approved the final manuscript.
